# Supplementary material for: Using A Low-Cost Sensor Array and Machine Learning Techniques to Detect Complex Pollutant Mixtures and Identify Likely Sources
Source: Sensors (Basel). 2019 Aug 28;19(17):3723. doi: 10.3390/s19173723 (PMC6749282; doi:10.3390/s19173723)
Supplement: Supplementary file 1 [file sensors-19-03723-s001.pdf]

SUPPLEMENTARY MATERIALS

Section S.1. Details of the Computational Methods as Applied Here

S.1.1. Validating Models

Because environmental data is highly autocorrelated, it was important to select holdout data for validation that would better represent unseen data than simple random sampling. For this study, holdout data was selected using k-fold validation with 10 folds as illustrated in Figure S1. Using this method, the full dataset was sorted by date and time and then divided into ten sequential blocks, or folds, of data. For all folds, each model was trained on the nine other folds of data and tested on the fold of interest. If model parameters were optimized for a given model, a subset of the training data was used for validation during that optimization process. The k-fold validation process produced ten estimates for each concentration and source at each timestep—one produced by each model trained on a subset of the data. This provided some indication of the sensitivity of the models to the set of data on which it was trained and, therefore, provides an indication of how well the model could generalize to new data relative to the other models.

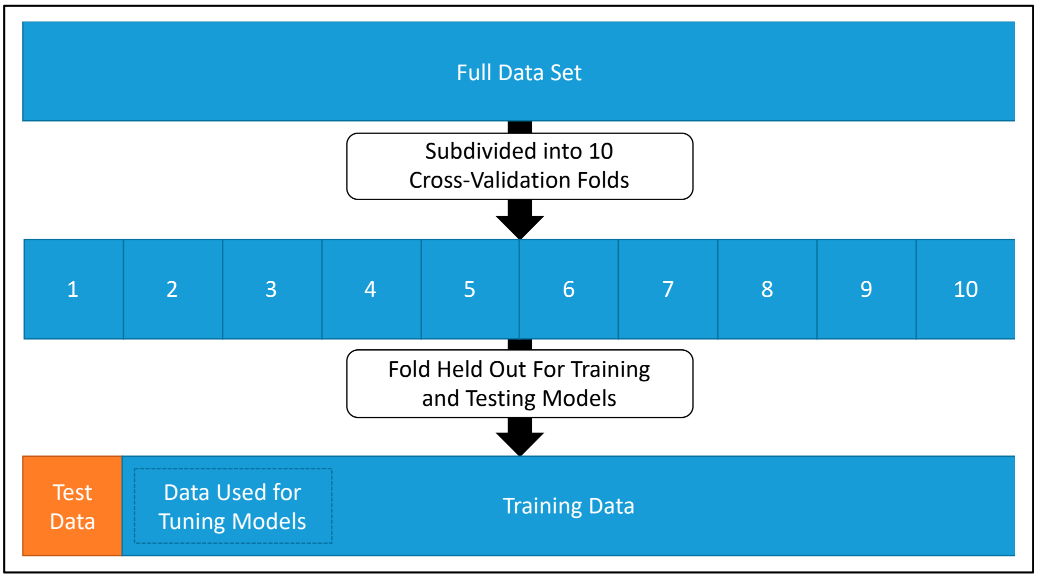

Figure S1. Graphic illustration of the 10-fold cross-validation method used here illustrating the use of the first fold for testing. Each fold contained 10% of the data, selected as consecutive blocks of time from start to end. Data used for validation when tuning hyperparameters were a subset of the training data (models were never exposed to the test data during training). For each cross-validation fold, classification models were tested on data that was also in the "test data" set during regression model training.

S.1.2. Estimating Concentrations with Regression Models

The first step in the analysis process involved regressing several "key" compounds that were selected as especially important for both detecting and differentiating the emissions from different classes of sources. Several regression techniques that have been applied in the literature were explored here and assessed first on their ability to accurately model the concentrations of several different gases. These regression techniques included multiple linear regression (MLR), ridge regression (RR), random forests (RF), gaussian process regression (GPR), and neural networks (NN). Separate models were trained for each

compound to avoid "learning" the artificial correlations that were present in this study but would not fully represent the diversity of mixtures that could be expected in a field deployment.

Some models include terms referred to here as "hyperparameters". Hyperparameters govern how the model operates or is trained and are set before beginning to train the model. For example, the number of layers and nodes in a neural network would be considered a hyperparameter. Another example is the regularization strength (k) in ridge regression, which modifies the loss function used while training the model. Tuning hyperparameters is one way to improve the performance of models and can be used to determine appropriate values for model parameters that might normally be selected arbitrarily or based on previous experience. Wherever possible, these hyperparameters were optimized to maximize the estimated model performance on future data. This was done by holding out a subset of the training data and optimizing the hyperparameters based on the model performance when validated on that subset of the training data. This data selection is illustrated in Figure S1. Once the hyperparameters were set, the regression models were also assessed based on how well the classification models in the next step were able to use the estimated concentrations to make a prediction of the "source" that was being simulated.

Multiple linear regression is one of the most popular forms of regression used to convert sensor signal values into calibrated concentration estimates and have been used in a range of applications with many low-cost sensor technologies [3,30,36,52–54]. Because of popularity and the relatively low computational costs, several forms of multiple linear regression were investigated. The first form, referred to here as "FullLM" was a multiple linear regression model that simply used every sensor input from the array. This was considered as a baseline model as it included almost no previous knowledge except for the design of the array itself. Next was a model, referred to as "SelectLM", included only data from sensors that were known to react to the current target gas, as determined by a combination of field experience and manufacturer recommendations. For each sensor included in a model, an interaction term between the sensor response and the measured temperature was included. Humidity was also included in each model, but no interaction term was added.

Stepwise linear regression ("StepLM") is an interesting but slow-to-train method of determining important predictors for use in a linear regression model. In this methodology, a base model (typically a simple intercept term or a full interaction model) is fit to the data. In the case of this study, the initial model was a simple intercept because a full interaction model was prohibitively large and slow. After fitting the original model, new models are trained with the addition or removal of one of each possible term. If the addition of a new term improves upon the previous model above some threshold, or if the removal does not reduce performance by a similar threshold, the term is added or removed. This new model is used as the new "base" model and the process is iterated until no terms can be added to or removed from the model within those constraints. In this study, the metric for improvement was the R^2 value, and the threshold was a value of 0.075, which was chosen by experimenting with different performance metrics and exploring the performance of the generated models.

Ridge regression ("RidgeLM") is a form of multiple linear regression, but in this study, the two are differentiated by how the terms of the multiple linear regression were determined. During the generation of ridge regression models, all sensor values were included, and additionally, the interaction between each sensor and both temperature and humidity were included. This created a high-dimensionality dataset, on which ridge regression was applied; a method that includes a term, "k", to penalize overfitting. Increasing the value of k affects the loss function, so that the final model assigns low weights to sensor signals that are not generally useful. This value of k was determined during initial investigations and then was kept constant across different compounds and cross-validation sets.

Outside of these linear regression models, several nonlinear models were trained. These were random forest regression ("RandFor"), gaussian process regression ("GuassProc"), and neural networks ("NeurNet"). For random forest regression, a large ensemble of decision trees are trained to output discrete values. These trees are each trained on different bootstrap aggregated ("bagged") subsets of the original

data that are selected randomly with replacement. When making predictions, the outputs of each of these trees are averaged in order to produce an output that can approximate a smooth function. Because the individual trees only learn to produce values that they have seen before, the extension of random forests outside of their training space may be limited, although Zimmerman and colleagues showed that they were able to produce reasonable results within some parameter space [65]. The hyperparameters that were optimized for random forests were the minimum number of points at each leaf node, the maximum number of splits for each tree, and the number of variables to select from at each edge. These were optimized using the loss function out-of-bag error, which is the error on data that were not selected during the "bagging" of data during initial training.

Gaussian process regression, which is sometimes referred to as kriging, is a probabilistic method that uses training data and some assumptions about the distribution of the variable to make predictions on new data. Because this method is nonparametric, the ability to extrapolate to new data is somewhat limited; however, it is popular in the environmental modeling community and De Vito recently showed good success applying them to real atmospheric data [53]. A squared exponential kernel was used after some initial investigation showed little dependence of the results on this selection. The hyperparameters that were programmatically optimized for this model were the kernel parameters and were optimized by minimizing the objective function: log(1 + cross-validation loss).

Finally, the last regression model explored here was a neural network, a technique that has been used with low-cost sensors for some time but is seeing a resurgence as improved training methods and computational power have improved their applicability [52,53,66]. These models produce results by combining a set of "neurons" into a larger network. Each neuron applies a set of weights to each input and uses a transfer function to translate the sum of those inputs into an output for the neuron. The first layer of neurons uses the raw sensor values as inputs, and subsequent layers use the outputs of the first layer as inputs. These two layers are often referred to as hidden layers, the last of which provides the input to the output layer that translates these outputs into a single predicted value. The hidden layers and number of nodes in each hidden layer of a neural network model are tunable hyperparameters and were optimized for the best mean squared error (MSE) on a subset of the training data that was held out for testing. The number of nodes in each hidden layer was varied between 1–40 for the first layer and from 0–40 for the second layer. When the number of nodes in the second layer was specified as "0", the second hidden layer was simply omitted.

S.2.3. Predicting the Presence of Sources with Classification Models

After generating estimates of "key" compounds using each of the above regression approaches, classification algorithms were trained to identify the class of "source" that was being simulated, using the estimated concentrations at each timestep as features. The data was divided into the same sets of calibration and validation sets to ensure that the final validation results were left out of model training for both regression and classification. The classification techniques applied here have been seen in the literature, although typically with the goal of identifying individual compounds within simple mixtures [10,55–57]. Those algorithms selected here are logistic regression, support vector machines (SVM) with both a linear and Gaussian kernel, random forest classifiers, and neural networks. The models vary significantly in their ability to separate different classes and were selected because of that diversity. For all of the methods presented here, the classification model outputs were values ranging from 0 to 1, where 0 indicates high confidence in the absence of a source, and 1 indicates high confidence in the presence of a source. When comparing the results to the actual simulated source, a value greater than or equal to 0.5 indicated a prediction that the source was present and a value less than 0.5 that it was absent.

The first type of classifier, the logistic regression, is the simplest and most linear of the classifiers. In the results below, this model was referred to as "Logistic_class" and is a generalized linear model with

binomial distribution. An independent classifier was created for each of the simulated sources, and each model was trained to indicate the presence or absence of that source. Much like the StepLM function described earlier, the terms for this model were selected by stepwise regression, with the difference being that the terms here were gas concentrations rather than sensor signal values. Because the logit link function is used to map the output of a linear function to a value from 0 to 1, the output of a logistic regression is often interpreted as the probability or confidence that the value is in the positive or negative class. In this case, that would be the likelihood that a certain source is or is not affecting the measured air quality. One benefit of logistic regression is that it is interpretable and computationally inexpensive relative to other, more complex and nonlinear methods.

The second classification model investigated was support vector machines (SVMs), which were trained to indicate the presence of a source. The general goal of SVMs is to create a line or plane that has the largest margin between separated classes, with some allowance for outliers and noise. In two-dimensional space, this can be visualized as creating a line that separates two classes and has the widest empty space on either side. The points closest to the line are referred to as support vectors and give the classifier its name. Because the goal of an SVM is to create a line that separates two classes, it may be considered as a linear classifier, although kernel functions are often used to map this linear function to a nonlinear space. The new features created by kernel methods are generally understood as measures of similarity between each instance. Two kernels were studied here, the first being a linear kernel that does not transform the input variables, and the second being the gaussian kernel. These are referred to in the results as "SVMlin_class" and "SVMgaus_class" respectively, simply because of the name of the functions written to implement them. Both kernels were implemented to predict the presence or absence of each source independently, just as described for logistic regression above. For the SVM with a gaussian kernel, the hyperparameters controlling the kernel scale and box constraint were optimized automatically to reduce cross-validated misclassification errors. These two factors affect how "smoothed" the kernel is and how heavily the loss function is penalized for errors, respectively.

Next, a random forest classification model was trained to identify the presence of each source, referred to in the results as "RandFor_class". Although random forests can quite easily be applied to multiclass regression problems, models were trained to predict the binary presence of each source separately, so that in sum, the models could predict the presence of multiple sources at the same time without creating many classes representing all possible combinations of sources. The hyperparameters that were optimized for this model were the same as for the random forest regression models: minimum leaf size and maximum number of splits. These random forests represent a collection of 500 decision trees that are each trained on different subsets of the training data. In this case, those subsets were selected via bootstrap aggregation (bagging), wherein data is randomly sampled with replacement from the original training dataset. Each of these trees produce a prediction of the presence or lack thereof for the source that they were trained on. The "score" that was output to indicate the confidence that a source was present represents the fraction of trees within each forest that predicted that a source was present.

The last classification approach that was explored was a pattern recognition neural network, referred to in the results as "NeurNet_class". This neural network had one output node for each source and was, therefore, able to predict the presence of multiple sources independently and simultaneously. The hyperparameters that were optimized for this model were the number of layers (1 or 2), the number of nodes in each layer, and the transfer function that translates the input to outputs. In this case, a neural net with two layers of five nodes using the "softmax" transfer function was selected.

Table S1. Table of sensors that were used for testing and the corresponding variable name(s) associated with those sensors.

Sensor Details	Variable Name(s)
Bosch BME 180 Barometric Pressure Sensor	bme_P
Sensirion Temperature and Humidity Sensor	temperature, humidity
ELT S300 NDIR CO_2 Sensor	co2_NDIR
Alphasense NO-B4 Sensor, Working and Auxiliary Electrode	NO_B4_aux, NO_B4_main
Alphasense NO2-B1 Sensor, Working and Auxiliary Electrode	NO2_B1_aux, NO2_B1_main
Alphasense CO-B4 Sensor, Working and Auxiliary Electrode	CO_B4_aux, CO_B4_main
Alphasense H2S-BH Sensor, Working and Auxiliary Electrode	H2S_BH_aux, H2S_BH_main
Alphasense O3-B4 Sensor, Working and Auxiliary Electrode	O3_B4_aux, O3_B4_main
Figaro 2600 Installed on the Pod Board in the 210 and 280 mW Heater Circuits, Sensing Voltage	fig2600_210ob_s, fig2600_280ob_s
Figaro 2602 Installed on the Pod Board in the 280 mW Heater Circuit, Sensing Voltage	fig2602_280ob_s
Baseline Mocon PID Sensor	bl_mocon
MICS-5121wp Installed on the Pod Board, Sensing Voltage	MICS5121wp_ob_s
MICS-2611 Installed on the Pod Board, Sensing Voltage	e2v2611_ob_s
MICS-2611 Installed on the External Board, Sensing Voltage	mics2611_s
MICS-2710 Installed on the External Board, Sensing Voltage	mics2710_s
MICS-5525 Installed on the External Board, Sensing Voltage	mics5525_s
MICS-5121wp Installed on the External Board, Sensing Voltage	mics5121wp_s
Figaro 4161 Installed on the External Board, Sensing Voltage	fig4161_sens
Figaro 2600 Installed on the External Board, Sensing Voltage	fig2600_sens
Figaro 2611 Installed on the External Board, Sensing Voltage	fig2611_sens
Figaro 2602 Installed on the External Board, Sensing Voltage	fig2602_sens

Table S2. Full list of test points with the mean values of temperature, humidity, and concentration that were recorded during that test point. The "Total" column indicates the total concentration of gases in the chamber, not including dilution gas. Some testing was limited by gas availability, which is why the quantity of test points for each source and concentration are not consistent.

Source	T (C)	RH (%)	Total (ppm)	NO_2 (ppm)	CO (ppm)	CO_2 (ppm)	CH_4 (ppm)	Ethane (ppm)	Propane (ppm)	Gasoline (ppm)
No Source	23	47	0.00	0.00	0.00	0	0.0	0.00	0.00	0.00
No Source	26	48	0.00	0.00	0.00	0	0.0	0.00	0.00	0.00
No Source	27	40	0.00	0.00	0.00	0	0.0	0.00	0.00	0.00
No Source	27	38	0.00	0.00	0.00	0	0.0	0.00	0.00	0.00
No Source	28	49	0.00	0.00	0.00	0	0.0	0.00	0.00	0.00
No Source	29	47	0.00	0.00	0.00	0	0.0	0.00	0.00	0.00
No Source	29	46	0.00	0.00	0.00	0	0.0	0.00	0.00	0.00
No Source	30	49	0.00	0.00	0.00	0	0.0	0.00	0.00	0.00
No Source	30	39	0.00	0.00	0.00	0	0.0	0.00	0.00	0.00
No Source	30	49	0.00	0.00	0.00	0	0.0	0.00	0.00	0.00
No Source	30	54	0.00	0.00	0.00	0	0.0	0.00	0.00	0.00
No Source	30	14	0.00	0.00	0.00	0	0.0	0.00	0.00	0.00
No Source	31	49	0.00	0.00	0.00	0	0.0	0.00	0.00	0.00
No Source	31	40	0.00	0.00	0.00	0	0.0	0.00	0.00	0.00
No Source	31	40	0.00	0.00	0.00	0	0.0	0.00	0.00	0.00
No Source	31	39	0.00	0.00	0.00	0	0.0	0.00	0.00	0.00
No Source	31	52	0.00	0.00	0.00	0	0.0	0.00	0.00	0.00
No Source	31	68	0.00	0.00	0.00	0	0.0	0.00	0.00	0.00
No Source	32	57	0.00	0.00	0.00	0	0.0	0.00	0.00	0.00
No Source	33	40	0.00	0.00	0.00	0	0.0	0.00	0.00	0.00
No Source	33	40	0.00	0.00	0.00	0	0.0	0.00	0.00	0.00
No Source	33	40	0.00	0.00	0.00	0	0.0	0.00	0.00	0.00
No Source	33	72	0.00	0.00	0.00	0	0.0	0.00	0.00	0.00
No Source	34	45	0.00	0.00	0.00	0	0.0	0.00	0.00	0.00
No Source	35	58	0.00	0.00	0.00	0	0.0	0.00	0.00	0.00
No Source	35	55	0.00	0.00	0.00	0	0.0	0.00	0.00	0.00
No Source	38	40	0.00	0.00	0.00	0	0.0	0.00	0.00	0.00
No Source	40	41	0.00	0.00	0.00	0	0.0	0.00	0.00	0.00
No Source	42	51	0.00	0.00	0.00	0	0.0	0.00	0.00	0.00
No Source	43	53	0.00	0.00	0.00	0	0.0	0.00	0.00	0.00
No Source	43	62	0.00	0.00	0.00	0	0.0	0.00	0.00	0.00
Gasoline Vapor	31	40	0.04	0.00	0.00	0	0.0	0.00	0.00	0.04
Gasoline Vapor	31	40	0.04	0.00	0.00	0	0.0	0.00	0.00	0.04

Source	T (C)	RH (%)	Total (ppm)	NO_2 (ppm)	CO (ppm)	CO_2 (ppm)	CH_4 (ppm)	Ethane (ppm)	Propane (ppm)	Gasoline (ppm)
Gasoline Vapor	36	40	0.04	0.00	0.00	0	0.0	0.00	0.00	0.04
Gasoline Vapor	39	41	0.04	0.00	0.00	0	0.0	0.00	0.00	0.04
Gasoline Vapor	40	40	0.04	0.00	0.00	0	0.0	0.00	0.00	0.04
Gasoline Vapor	40	42	0.04	0.00	0.00	0	0.0	0.00	0.00	0.04
Gasoline Vapor	31	40	0.11	0.00	0.00	0	0.0	0.00	0.00	0.11
Gasoline Vapor	32	40	0.11	0.00	0.00	0	0.0	0.00	0.00	0.11
Gasoline Vapor	38	40	0.11	0.00	0.00	0	0.0	0.00	0.00	0.11
Gasoline Vapor	39	41	0.11	0.00	0.00	0	0.0	0.00	0.00	0.11
Gasoline Vapor	40	41	0.11	0.00	0.00	0	0.0	0.00	0.00	0.11
Gasoline Vapor	40	40	0.11	0.00	0.00	0	0.0	0.00	0.00	0.11
Gasoline Vapor	31	40	0.24	0.00	0.00	0	0.0	0.00	0.00	0.24
Gasoline Vapor	32	40	0.24	0.00	0.00	0	0.0	0.00	0.00	0.23
Gasoline Vapor	40	40	0.23	0.00	0.00	0	0.0	0.00	0.00	0.23
Gasoline Vapor	40	41	0.24	0.00	0.00	0	0.0	0.00	0.00	0.24
Gasoline Vapor	40	40	0.24	0.00	0.00	0	0.0	0.00	0.00	0.24
Gasoline Vapor	40	41	0.24	0.00	0.00	0	0.0	0.00	0.00	0.23
Gasoline Vapor	26	49	0.35	0.00	0.00	0	0.0	0.00	0.00	0.35
Gasoline Vapor	27	50	0.30	0.00	0.00	0	0.0	0.00	0.00	0.30
Gasoline Vapor	29	46	0.31	0.00	0.01	0	0.0	0.00	0.00	0.30
Gasoline Vapor	29	49	0.35	0.00	0.00	0	0.0	0.00	0.00	0.35
Gasoline Vapor	31	50	0.30	0.00	0.00	0	0.0	0.00	0.00	0.29
Gasoline Vapor	31	40	0.31	0.00	0.00	0	0.0	0.00	0.00	0.10
Gasoline Vapor	29	47	0.35	0.00	0.01	0	0.0	0.00	0.00	0.35
Gasoline Vapor	31	40	0.44	0.00	0.00	0	0.0	0.00	0.00	0.44
Gasoline Vapor	38	65	0.44	0.00	0.00	0	0.0	0.00	0.00	0.44
Gasoline Vapor	32	40	0.48	0.00	0.00	0	0.0	0.00	0.00	0.48
Gasoline Vapor	40	41	0.48	0.00	0.00	0	0.0	0.00	0.00	0.48
Gasoline Vapor	40	40	0.48	0.00	0.00	0	0.0	0.00	0.00	0.48
Gasoline Vapor	42	53	0.79	0.00	0.00	1	0.0	0.00	0.00	0.10
Heavy Exhaust	37	73	57.87	0.69	0.56	57	0.0	0.00	0.00	0.00
Heavy Exhaust	31	40	60.07	0.69	0.57	59	0.0	0.00	0.00	0.00
Heavy Exhaust	41	67	145.92	1.40	0.27	144	0.0	0.00	0.00	0.00
Heavy Exhaust	43	62	145.94	0.33	1.14	144	0.0	0.00	0.00	0.00
Heavy Exhaust	32	40	145.99	0.33	1.15	145	0.0	0.00	0.00	0.00
Heavy Exhaust	42	61	146.08	1.40	0.56	144	0.0	0.00	0.00	0.00
Heavy Exhaust	43	62	146.19	0.69	1.14	144	0.0	0.00	0.00	0.00

Source	T (C)	RH (%)	Total (ppm)	NO_2 (ppm)	CO (ppm)	CO_2 (ppm)	CH_4 (ppm)	Ethane (ppm)	Propane (ppm)	Gasoline (ppm)
Heavy Exhaust	32	40	146.37	0.69	1.15	145	0.0	0.00	0.00	0.00
Heavy Exhaust	31	40	146.87	1.40	0.57	145	0.0	0.00	0.00	0.00
Heavy Exhaust	40	69	146.91	1.40	1.14	144	0.0	0.00	0.00	0.00
Heavy Exhaust	31	40	147.18	1.40	0.28	146	0.0	0.00	0.00	0.00
Heavy Exhaust	31	40	148.38	1.40	1.15	146	0.0	0.00	0.00	0.00
Heavy Exhaust	38	55	195.05	0.69	0.57	194	0.0	0.00	0.00	0.00
Heavy Exhaust	31	40	195.53	0.69	0.57	194	0.0	0.00	0.00	0.00
Heavy Exhaust	39	40	195.51	0.69	0.57	194	0.0	0.00	0.00	0.00
Heavy Exhaust	33	61	281.17	0.33	1.15	280	0.0	0.00	0.00	0.00
Heavy Exhaust	39	48	282.55	1.40	0.28	281	0.0	0.00	0.00	0.00
Heavy Exhaust	31	40	282.86	1.40	0.28	281	0.0	0.00	0.00	0.00
Heavy Exhaust	39	46	282.91	1.40	0.57	281	0.0	0.00	0.00	0.00
Heavy Exhaust	39	44	282.90	0.33	1.15	281	0.0	0.00	0.00	0.00
Heavy Exhaust	39	45	283.02	0.69	1.15	281	0.0	0.00	0.00	0.00
Heavy Exhaust	31	40	283.13	0.69	1.15	281	0.0	0.00	0.00	0.00
Heavy Exhaust	31	40	283.17	1.40	0.57	281	0.0	0.00	0.00	0.00
Heavy Exhaust	39	51	283.28	1.40	1.15	281	0.0	0.00	0.00	0.00
Heavy Exhaust	39	40	283.46	1.40	0.28	282	0.0	0.00	0.00	0.00
Heavy Exhaust	38	40	283.56	0.33	1.15	282	0.0	0.00	0.00	0.00
Heavy Exhaust	31	40	283.86	1.40	1.15	281	0.0	0.00	0.00	0.00
Heavy Exhaust	39	40	283.92	0.69	1.15	282	0.0	0.00	0.00	0.00
Heavy Exhaust	39	40	283.94	1.40	0.57	282	0.0	0.00	0.00	0.00
Heavy Exhaust	39	40	284.37	1.40	1.15	282	0.0	0.00	0.00	0.00
Low T Combustion	29	49	0.44	0.00	0.44	0	0.0	0.00	0.00	0.00
Low T Combustion	30	49	0.45	0.00	0.45	0	0.0	0.00	0.00	0.00
Low T Combustion	30	50	0.36	0.00	0.36	0	0.0	0.00	0.00	0.00
Low T Combustion	31	40	0.45	0.00	0.45	0	0.0	0.00	0.00	0.00
Low T Combustion	40	60	0.45	0.00	0.45	0	0.0	0.00	0.00	0.00
Low T Combustion	31	40	103.54	0.00	0.00	104	0.0	0.00	0.00	0.00
Low T Combustion	36	60	103.78	0.00	0.00	104	0.0	0.00	0.00	0.00
Low T Combustion	38	40	103.93	0.00	0.00	104	0.0	0.00	0.00	0.00
Low T Combustion	39	40	104.02	0.00	0.00	104	0.0	0.00	0.00	0.00
Low T Combustion	30	49	564.64	0.00	0.00	565	0.0	0.00	0.00	0.00
Low T Combustion	41	56	567.67	0.00	0.01	568	0.0	0.00	0.00	0.00
Low T Combustion	31	40	570.85	0.00	0.01	571	0.0	0.00	0.00	0.00
Natural Gas	30	40	1.49	0.00	0.00	0	1.5	0.00	0.00	0.00

Source	T (C)	RH (%)	Total (ppm)	NO_2 (ppm)	CO (ppm)	CO_2 (ppm)	CH_4 (ppm)	Ethane (ppm)	Propane (ppm)	Gasoline (ppm)
Natural Gas	30	39	1.49	0.00	0.00	0	1.5	0.00	0.00	0.00
Natural Gas	31	40	1.50	0.00	0.00	0	1.5	0.00	0.00	0.00
Natural Gas	34	40	1.49	0.00	0.00	0	1.5	0.00	0.00	0.00
Natural Gas	34	45	1.49	0.00	0.00	0	1.5	0.00	0.00	0.00
Natural Gas	40	58	1.51	0.00	0.00	0	1.5	0.00	0.00	0.00
Natural Gas	30	39	1.92	0.00	0.00	0	1.5	0.43	0.00	0.00
Natural Gas	30	40	1.92	0.00	0.00	0	1.5	0.43	0.00	0.00
Natural Gas	31	40	1.92	0.00	0.00	0	1.5	0.43	0.00	0.00
Natural Gas	32	56	1.92	0.00	0.00	0	1.5	0.43	0.00	0.00
Natural Gas	32	52	1.92	0.00	0.00	0	1.5	0.43	0.00	0.00
Natural Gas	34	40	1.93	0.00	0.00	0	1.5	0.43	0.00	0.00
Natural Gas	42	55	1.92	0.00	0.00	0	1.5	0.42	0.00	0.00
Natural Gas	30	40	3.29	0.00	0.00	0	3.3	0.00	0.00	0.00
Natural Gas	30	39	3.28	0.00	0.00	0	3.3	0.00	0.00	0.00
Natural Gas	34	40	3.28	0.00	0.00	0	3.3	0.00	0.00	0.00
Natural Gas	34	46	3.27	0.00	0.00	0	3.3	0.00	0.00	0.00
Natural Gas	35	47	3.28	0.00	0.00	0	3.3	0.00	0.00	0.00
Natural Gas	30	40	4.18	0.00	0.00	0	3.3	0.90	0.01	0.00
Natural Gas	31	39	4.18	0.00	0.00	0	3.3	0.90	0.01	0.00
Natural Gas	33	52	4.18	0.00	0.00	0	3.3	0.90	0.01	0.00
Natural Gas	33	50	4.18	0.00	0.00	0	3.3	0.90	0.01	0.00
Natural Gas	34	40	4.19	0.00	0.00	0	3.3	0.90	0.01	0.00
Natural Gas	30	40	13.93	0.00	0.00	0	13.9	0.01	0.00	0.00
Natural Gas	30	39	13.92	0.00	0.00	0	13.9	0.00	0.00	0.00
Natural Gas	31	40	13.92	0.00	0.00	0	13.9	0.01	0.00	0.00
Natural Gas	34	40	13.94	0.00	0.00	0	13.9	0.01	0.00	0.00
Natural Gas	34	47	13.93	0.00	0.00	0	13.9	0.01	0.00	0.00
Natural Gas	35	48	13.94	0.00	0.00	0	13.9	0.00	0.00	0.00
Natural Gas	38	62	13.91	0.00	0.00	0	13.9	0.00	0.00	0.00
Natural Gas	31	40	17.65	0.00	0.00	0	13.9	3.74	0.05	0.00
Natural Gas	43	52	17.64	0.00	0.00	0	13.9	3.72	0.05	0.00
Natural Gas	30	40	17.68	0.00	0.00	0	13.9	3.74	0.07	0.00
Natural Gas	30	39	17.71	0.00	0.00	0	13.9	3.75	0.07	0.00
Natural Gas	34	48	17.68	0.00	0.00	0	13.9	3.74	0.07	0.00
Natural Gas	34	40	17.69	0.00	0.00	0	13.9	3.74	0.07	0.00
Natural Gas	34	49	17.69	0.00	0.00	0	13.9	3.74	0.07	0.00

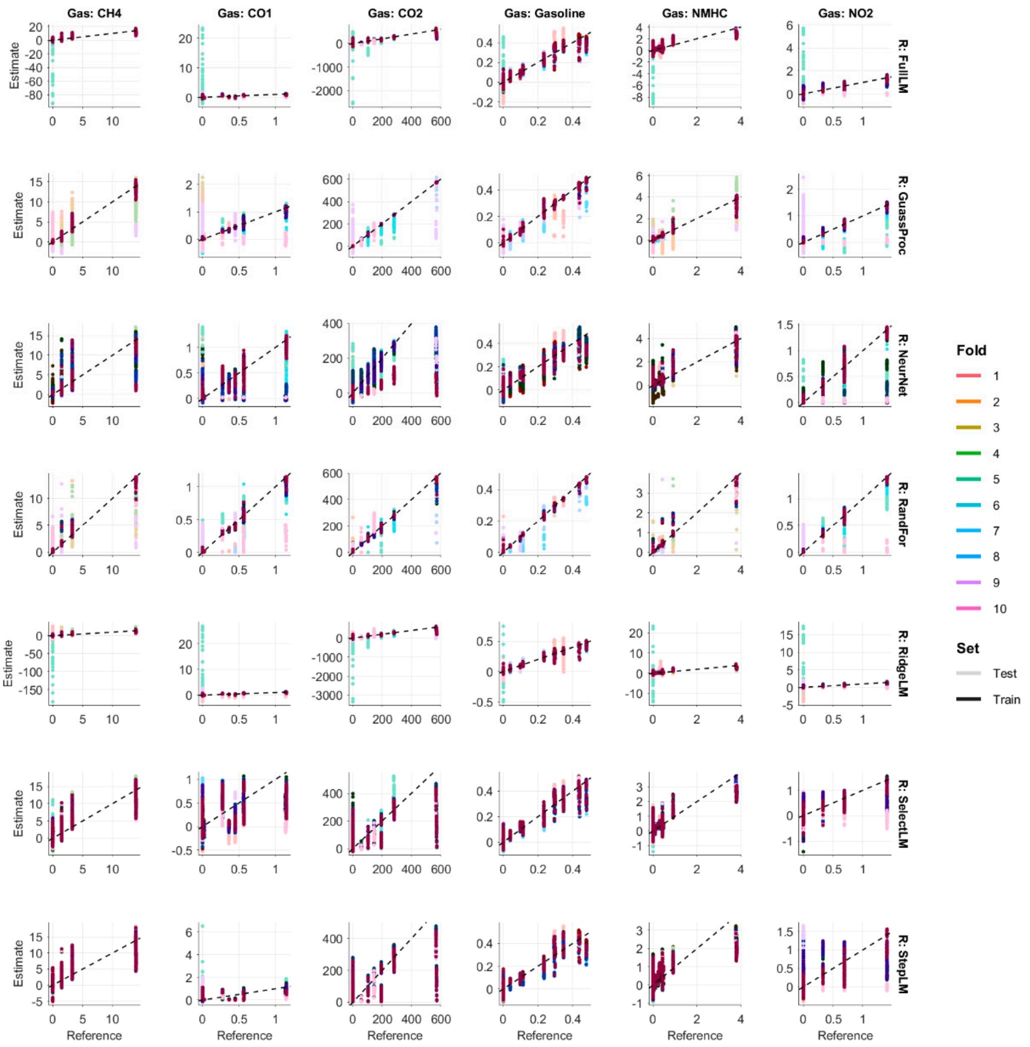

Figure S2. Estimated versus reference concentrations are plotted on the next page for each combination of gas and regression technique. Each column of plots contains estimates for a given gas, and each row contains estimates for a given regression technique. The color of each point indicates the cross-validation fold that was used for training and testing the model, and the shade (light versus dark) indicate whether the values were estimated on training data or testing (validation) data.
